# An Immunological Non-responder Human Immunodeficiency Virus/Hepatitis C Virus Coinfected Patient: Considerations About a Clinical Case

**DOI:** 10.7759/cureus.37063

**Published:** 2023-04-03

**Authors:** Rui Soares Correia, Margarida França

**Affiliations:** 1 Department of Internal Medicine, Centro Hospitalar Tondela-Viseu - Epe, Viseu, PRT; 2 Department of Internal Medicine, Centro Hospitalar do Porto, Porto, PRT

**Keywords:** co-infection, immunological reconstitution syndrome, antiretroviral therapy, hepatitis c virus, human immunodeficiency virus

## Abstract

Human immunodeficiency virus (HIV) and hepatitis C virus (HCV) infections are two chronic viral infections that share the same mode of transmission, making HIV/HCV coinfection frequent. Highly active antiretroviral therapy (HAART) was a turning point in HIV treatment and has been shown to successfully restore immune function and reduce the frequency of opportunistic infections. Despite a virological response to HAART, a proportion of patients fail to achieve substantial immune recovery, as measured by peripheral CD4 cell counts.

Herein, we present the case of a patient with HIV/HCV coinfection who did not achieve successful immune function restoration despite HIV suppression and HCV treatment.

Our goal is to promote discussion. Despite considerable advances in the understanding of the impact of HCV on HIV disease progression, there are many individual variables that influence a patient’s immune function. In addition, we consider hypogammaglobulinemia as a possible contributor. Further understanding and improvement of immune reconstitution in patients infected with HIV remain an important field of scientific research.

## Introduction

Human immunodeficiency virus (HIV) and hepatitis C virus (HCV) infections are chronic viral infections that affect millions of people worldwide. People who inject drugs accounted for 5%, while men who have sex with men accounted for 81% of the new HIV diagnoses in the United States in 2018 [1]. These two populations also report a high prevalence of co-infection with HCV (median of 3.9% in Europe), as HIV and HCV share the same mode of transmission [2].

The development of highly active antiretroviral therapy (HAART) was a turning point in HIV treatment, substantially improving HIV management. HAART has been shown to successfully restore immune function and reduce opportunistic infections. This restoration of immune function markedly improves HIV-associated diseases and reduces acquired immunodeficiency syndrome (AIDS)-related mortality [3,4].

Currently, there is no consensus on the definition of immunological non-responders (INRs). Over the years, INRs have been described as individuals who do not reach pre-defined CD4 cell counts despite full viral suppression on HAART [5]. Depending on the definition, 10-40% of patients with HIV infection are classified as INRs, despite the virological response to HAART. These patients have an increased risk of AIDS progression [6].

## Case presentation

A 46-year-old man with a history of injecting drug usage was diagnosed with genotype 1 HCV infection during an episode of jaundice in 2002. The patient was negative for HIV infection until 12 months after the initial presentation, when HIV-1 infection was diagnosed. At the time, he presented with a CD4 cell count of 393 cells/µL and an HIV viremia of 12,113 copies/µL; however, he did not meet the treatment criteria for either disease. He was lost to follow-up in 2004.

**Figure 1:**
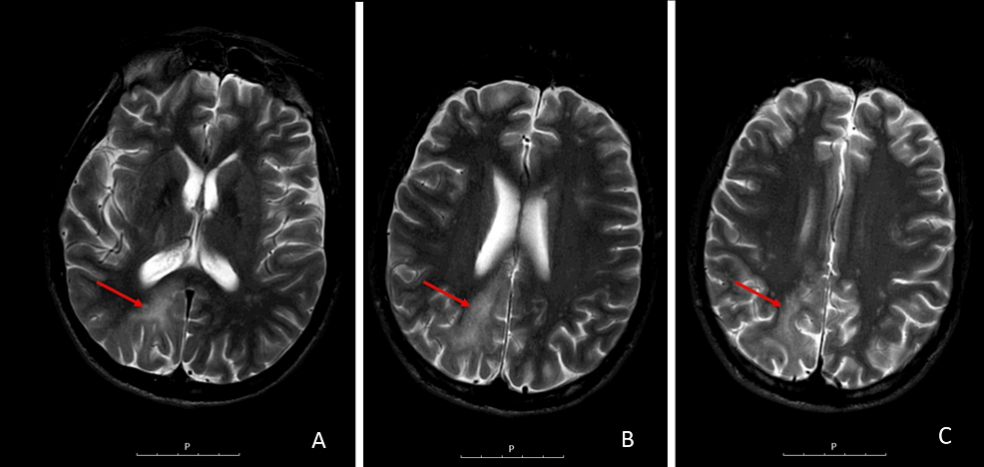
Cerebral MRI axial sections showing a right occipito-parietal lesion with hyperintense signal in T2-weighted sequence, suggesting progressive multifocal leukoencephalopathy (arrows) MRI: magnetic resonance imaging

In 2011, the patient was admitted to the hospital owing to recurrent fever and was diagnosed with ganglionic tuberculosis (TB). At this time, he had a CD4 cell count of 16 cells/µL (4%), and an HIV viremia of 107,000 copies/µL. Tenofovir, emtricitabine, nevirapine, and prophylactic cotrimoxazole were initiated, with the addition of quadruple antimycobacterial therapy one week later in an outpatient setting. One month after starting HAART, the patient was re-admitted to the hospital with worsening TB in the setting of immune reconstitution inflammatory syndrome (IRIS). He had a CD4 cell count of 75 cells/µL (11%), and an HIV viremia of 158 copies/µL. Corticosteroid therapy was initiated. Owing to clinical improvement, he was discharged after two weeks. During an outpatient consultation two months after starting HAART, he was lethargic, with left-sided weakness, dysmetria, and hemispatial neglect. Cerebrospinal fluid analysis showed an HIV viremia of 4,340 copies/µL and a positive PCR JC virus. Cerebral magnetic resonance imaging revealed a right occipito-parietal lesion with a hyperintense signal in T2-weighted and FLAIR MRI sequences, suggesting the diagnosis of progressive multifocal leukoencephalopathy (Figure 1).

HAART was switched to zidovudine, abacavir, and nevirapine, while antimycobacterial therapy was adjusted to isoniazid and rifampicin. Consequently, there was a progressive regression of the neurologic findings, and he was discharged asymptomatic after two weeks in the hospital. He was maintained on antimycobacterial therapy for one year.

After six months on antiretroviral therapy (ART), HIV-1 was suppressed (HIV viremia of <20 copies/µL); however, the patient maintained a CD4 cell count of <100 cells/µL. Accordingly, an HIV drug-resistance test was performed in 2012, which revealed possible resistance to zidovudine, efavirenz, and etravirine and confirmed resistance to nevirapine. HAART was then adjusted to abacavir, raltegravir, and darunavir/ritonavir.

The patient maintained virological suppression since 2012, while no immunological recovery was achieved (consistent CD4 cell count of <100 cells/µL [8%]). The immunoglobulin assay indicated low IgG levels without clinical or laboratory criteria for immunoglobulin replacement therapy. At the time of the HIV infection diagnosis, IgG levels were normal. In 2016, a lymphocytic study was performed, which concluded that 22.1% of the patient’s peripheral B lymphocytes had CD31 and CD45RA staining, indicating a decrease in thymic output.

From the perspective of HCV treatment, ART was changed to abacavir, lamivudine, and dolutegravir in 2017. In 2018, HCV hepatitis was treated with elbasvir and grazoprevir for 16 weeks with virological remission. At the time of submission of this article, the patient had undetectable HIV viremia and a CD4 cell count consistently below 100 cells/µL (<12%).

## Discussion

Multiple factors shape the natural history of HIV. In this case, there was an IRIS event associated with ganglionic TB treatment, and we speculate that IRIS events predict immunological reconstitution. Despite the recent advances in the understanding of IRIS pathogenesis and the numerous studies demonstrating the key role of T helper 1 CD4 cells in mycobacterial IRIS [7,8], there is no consensus, as previous findings suggest that inflammatory pathways in addition to an increased number of CD4 cells are involved in the development of IRIS [9]. Therefore, there is no clear association between IRIS and immunological reconstitution.

Several immunopathogenic mechanisms have been reported to be associated with incomplete immune reconstitution; however, none of these alone can fully explain this phenomenon, which is most likely multifactorial [10].

Immunopathogenic mechanisms are associated with decreased CD4 cell production. HIV can infect bone marrow CD34+ hematopoietic progenitor cells; as T cells originate from these cells, impaired bone marrow hematopoietic function and decreased proliferative capacity might be associated with incomplete immune reconstitution in individuals infected with HIV-1 [11]. The thymic function is also essential for the generation of naïve CD4 and CD8 cells with a broad T-cell receptor repertoire [4]. Herein, the patient had decreased thymic output. Recent studies have concluded that reduced thymic function, especially when associated with cell death by pyroptosis, is the major mechanism of immunological recovery failure in HAART-treated patients [12]. Furthermore, studies have shown that perturbations in cytokine secretion play a key role in immunologic recovery. Interleukin-7 (IL-7), produced by stromal cells in lymphoid organs, is crucial for CD4 cell homeostasis, promoting the survival, proliferation, and production of these cells. The pro-inflammatory state and infiltration of lymphoid tissues by HIV-1 result in progressive collagen deposition in these tissues, limiting IL-7 secretion [13,14].

Other immunopathogenic mechanisms are associated with CD4 cell destruction. These mechanisms are based on the persistent activation of the immune system that occurs in HIV-1 infection. Although ART reduces the level of immune activation and inflammation, it fails to normalise the activation of CD4 cells [15]. The upregulation of immune checkpoint receptors by the persistent activation of the immune system is associated with T-cell exhaustion, which is characterised by the decreased proliferation and production of cytokines [16]. Furthermore, gut barrier disruption is observed in the early stages of HIV-1 infection as massive amounts of CD4 cells in the gut are depleted. These abnormalities eventually result in the alteration of the intestinal microbiota composition and the release of bacterial products into the circulation, leading to chronic immune activation and inflammation [17]. Furthermore, as in this patient, coinfections, namely the HCV coinfection, must be considered. Potter et al. demonstrated that individuals with HIV/HCV coinfection and persistent HCV RNA viremia have a slower recovery of CD4 cells on HAART than individuals with cleared HCV RNA viremia, suggesting that active HCV infection influences immune restoration [18]. HCV infection affects the liver, which is a lymphoid organ essential for maintaining T-cell homeostasis and is associated with a permanent state of immune activation that favours the destruction of CD4 cells [19].

There are many other factors that influence immune reconstitution in individuals infected with HIV-1, such as older age, male sex, low nadir CD4 cell count, low CD4/CD8 ratio, and low naïve/memory CD4 cell ratio [10].

We also attempted to elucidate the role of hypogammaglobulinemia in non-immunological recovery; however, literature on this subject is scarce. Most studies have been conducted in children and reported that most children infected with HIV have hypergammaglobulinemia, which may be the earliest manifestation of HIV infection in vertically exposed infants [20]. However, even in those with hypergammaglobulinemia, specific antibody responses are often subnormal. They may, like patients with hypogammaglobulinemia, have an increased susceptibility to bacterial infections and benefit from intravenous immunoglobulin [21].

## Conclusions

Despite marked advances in the understanding of the impact of HCV on HIV disease progression, there are many variables affecting individual immunological recovery. Moreover, different patients may have different dominant mechanisms that are responsible for poor immune reconstitution. Further understanding and improvement of immune reconstitution in patients infected with HIV-1 remain important fields of scientific research.
